# Workplace social capital and turnover intention in nursing home caregivers: the mediating role of occupational burnout and the moderating role of professional certification

**DOI:** 10.3389/fpsyg.2026.1822004

**Published:** 2026-07-29

**Authors:** Qiujie Zhang, Zhaoxi Han, Qinghui Peng

**Affiliations:** 1 Beijing Vocational College of Labor and Social Security, Beijing, China; 2 Peking University Sustainability Research Institute, Jinan, China

**Keywords:** nursing home caregivers, occupational burnout, professional certification, turnover intention, workplace social capital

## Abstract

This cross-sectional study examined correlational associations among workplace social capital, occupational burnout, professional certification, and turnover intention in a sample of 222 Chinese nursing home caregivers, grounded in Social Capital Theory and Conservation of Resources Theory. Covariate-adjusted correlational results indicated a negative concurrent linkage between workplace social capital and turnover intention, with occupational burnout exhibiting a statistical mediating pattern in this linkage (indirect association = −0.0917, 95% bias-corrected bootstrap CI [−0.1883, −0.0199]). Professional certification functioned as a significant first-stage moderator of this statistical indirect pathway (index of moderated mediation = 0.1527, 95% bias-corrected bootstrap CI [0.0151, 0.3180]). The statistical indirect association via occupational burnout reached significance only among uncertified caregivers, while the corresponding indirect linkage was non-significant for certified caregivers. Higher concurrent workplace social resources corresponded to weaker burnout and turnover intention to a stronger degree for caregivers lacking professional certification with limited personal human capital endowments. This study integrates Social Capital Theory and Conservation of Resources Theory to delineate differentiated correlational patterns across caregiver subgroups, and puts forward targeted retention strategies differentiated by certification background for long-term care facilities.

## 1 Introduction

Turnover is an acute and prevalent challenge for nursing home caregivers worldwide (Marmondi et al., 2022). In China, studies indicate that the overall turnover rate for caregivers in nursing homes is approximately 50% (Zeng et al., 2024). High turnover co-occurs with loss of professional human resources and detrimental organizational outcomes, such as reduced efficiency (Krsnik and Erjavec, 2023), unstable service quality, and nursing shortage (Boamah et al., 2022).

Studies to date have identified several antecedents of turnover intention, including organizational culture, perceived supervisor support, organizational commitment, and perceived organizational justice (Bakker and Demerouti, 2017; Zhu et al., 2022). However, Existing research separately examines fragmented workplace factors, and few studies treat workplace social capital as a unified construct, leaving its overall correlational role underexplored (Zhang et al., 2022). Consequently, the holistic role of workplace social capital (WSC) as a unified construct remains underexplored in the turnover literature (Zhang et al., 2022). Moreover, no study has specifically examined this construct in the context of nursing home caregivers, a population that faces especially high emotional demands and retention challenges.

To address these gaps, this study investigates the association between WSC and turnover intention in nursing home caregivers in China. It advances our theoretical understanding by treating WSC as a coherent, composite construct rather than a collection of fragmented elements. It also extends the empirical application of this construct to a critically understudied yet vital segment of the healthcare workforce.

## 2 Theory and hypotheses

The sustainability of nursing home caregivers has emerged as a critical public health challenge, particularly in aging societies like China. With over 310 million individuals aged 60 or above, accounting for nearly 22% of the total population, China’s demand for elderly care services has surged dramatically (Ministry of Civil Affairs of China, 2024). Unfortunately, this growing need is paired with a shrinking and increasingly unstable workforce.

### 2.1 Turnover intention

Turnover intention is the cognitive precursor to actual job exit (Mobley, 1977). It is a central focus of human resource research because of its strong predictive validity for real-world attrition (Tham, 2007). In the context of elderly care, characterized by a high degree of emotional labor and physical demands, understanding correlates of turnover intention is essential for developing targeted retention strategies. Traditionally, economic compensation and improved working conditions have been emphasized as possible approaches, but recent scholarship has revealed the importance of psychosocial factors such as workplace relationships, organizational support, and mental well-being (Ren, 2021).

### 2.2 Workplace social capital: a protective organizational resource

Workplace social capital is the collective value an employee derives from interpersonal networks, shared norms, trust, reciprocity, and cooperation in an organizational setting (Liu et al., 2025). It encompasses three interrelated dimensions: bonding (strong ties among peers), bridging (weaker but cross-group connections), and linking (vertical relationships between employees and management) (Park et al., 2024). Higher WSC scores co-occur with greater access to interpersonal support resources (Seibert et al., 2001). High levels of WSC signify harmonious relationships, enhanced trust, and mutual commitment, contributing to an improved quality of work life. Scholars have found that WSC is associated with employees’ turnover and turnover intentions (Zhang et al., 2022, 2025). Empirical evidence consistently supports the protective association of WSC against adverse employment outcomes (Aarons et al., 2015; Jun et al., 2023). These findings agree with Social Capital Theory, which posits that access to relational resources enables individuals to mobilize instrumental and emotional support that enhances their capacity to navigate workplace challenges (Putnam, 2000).

WSC is especially vital in the high-pressure environment of elderly care. High levels of WSC are correlated with nursing home caregivers’ access to valuable resources, such as practical work strategies, professional development opportunities, and emotional support, as well as a lower likelihood of turnover intention. WSC exhibits a concurrent negative statistical linkage with stress and turnover intention. Accordingly, we propose as follows:

*Hypothesis 1 (H1)*: Workplace social capital is negatively associated with turnover intention in nursing home caregivers.

### 2.3 Occupational burnout: a key psychological mechanism

Occupational burnout, defined as a syndrome of emotional exhaustion, depersonalization, and reduced personal accomplishment (Kuriyama et al., 2023), is one of the most prevalent occupational health concerns in the global healthcare sector. Burnout is typically viewed as a state of resource depletion (Barthauer et al., 2020), with empirical evidence demonstrating its negative association with workplace social capital (Loghman et al., 2023) and its robust positive association with turnover intention (Hsu and Yang, 2022; Tu et al., 2025). Prolonged emotional labor co-occurs with higher burnout levels among caregivers (Liu et al., 2025).

Conservation of Resources (COR) Theory (Hobfoll, 1989; Hobfoll et al., 2018) provides the theoretical lens for understanding these relationships. COR theory posits that individuals strive to obtain, retain, and protect valued resources; when resource loss exceeds resource gain, stress and burnout occur. In this context, WSC displays negative concurrent correlations with resource depletion, burnout, and turnover intention in sequence. Therefore, we propose:

*Hypothesis 2 (H2)*: Occupational burnout exhibits a statistical mediating correlational pattern in the cross-sectional association between workplace social capital and turnover intention.

### 2.4 The moderating role of professional certification

While WSC functions as an environmental resource, the extent to which employees benefit from it may depend on their personal resource endowments. Professional certification—a credential indicating formal competence and human capital—is one such personal resource that may shape how caregivers perceive and mobilize workplace social capital (Becker, 1964).

Integrating Social Capital Theory (Putnam, 2000) and COR Theory (Hobfoll, 1989; Hobfoll et al., 2018), we conceptualize WSC as an environmental resource that correlates with greater access to social support and lower levels of resource depletion, while professional certification serves as a personal resource endowment that moderates this resource-buffering process. Specifically, certified caregivers possess greater human capital, which may reduce their reliance on workplace social capital to mitigate burnout and turnover intention. Non-certified caregivers, lacking such credential-based advantages, may depend more heavily on workplace relational resources as a compensatory mechanism. This logic is consistent with COR theory’s notion of “resource caravans”—individuals with greater initial resources are better positioned to acquire and protect additional resources, while those with fewer resources are more vulnerable to resource loss spirals (Hobfoll et al., 2018).

In the context of Chinese nursing homes, where disparities in professional status and compensation between certified and non-certified caregivers are pronounced, professional certification likely serves as a pivotal moderating mechanism. Accordingly, we propose:

*Hypothesis 3 (H3)*: Professional certification moderates the first stage of the indirect relationship between workplace social capital and turnover intention via occupational burnout. Specifically, the negative association between workplace social capital and occupational burnout is stronger for non-certified caregivers.

The inclusion of this moderating variable makes a more nuanced examination of the underlying dynamics possible. Specifically, it allows us to investigate whether nursing home caregivers possessing vocational skill certifications differ from those without such credentials in terms of how workplace social capital is associated with their turnover intentions. This, in turn, yields valuable insights into the potential role of professional certification in shaping nursing home caregivers’ work-related attitudes and behaviors, offering implications for targeted interventions and policymaking aimed at mitigating turnover rates in this sector.

**Figure 1:**
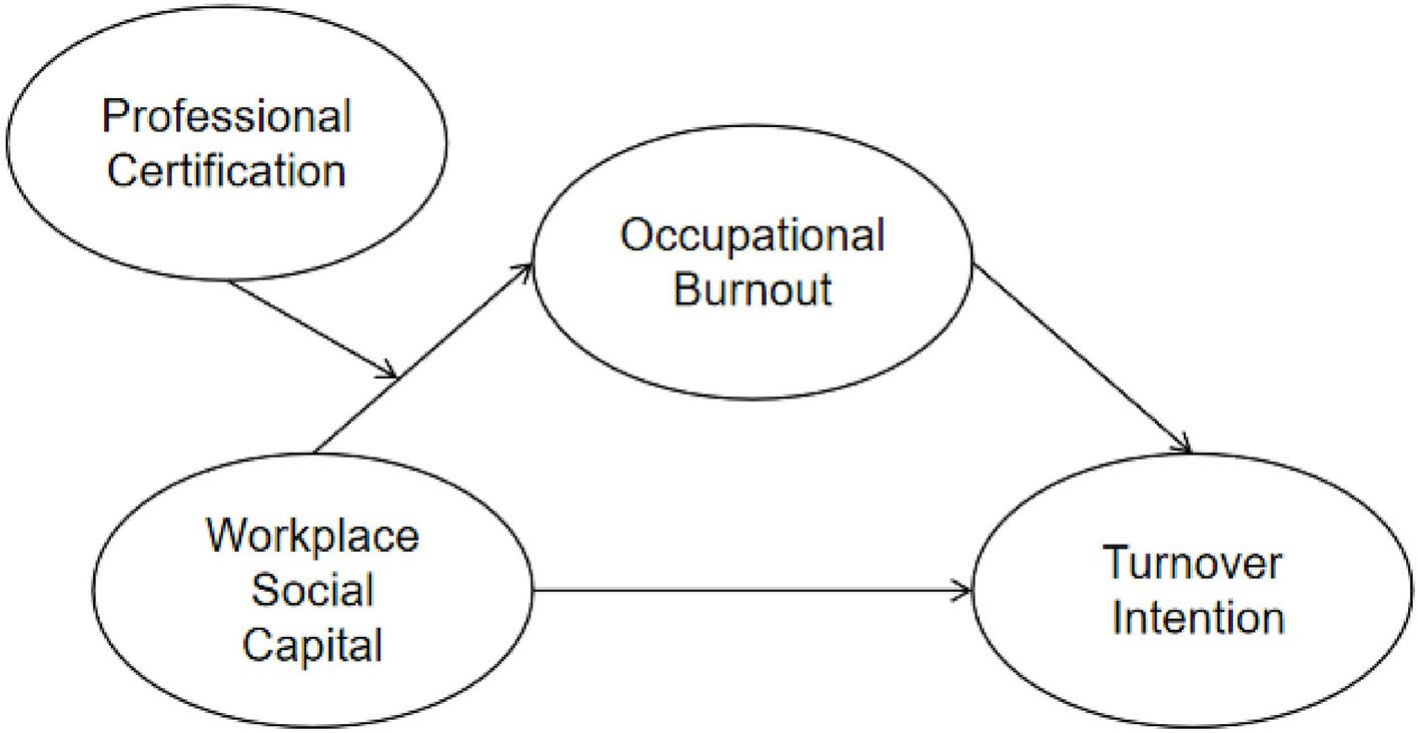
Theoretical first-stage moderated mediation model. Professional certification moderates the path from workplace social capital to occupational burnout (the first stage of mediation).

In summary, this study constructs a theoretical model of the influence mechanism by which WSC is associated with caregiver turnover intention, incorporating occupational burnout as a mediator and professional certification as a moderator, as shown in Figure 1.

## 3 Methods

### 3.1 Participants

Frontline caregivers employed in nursing homes across the Beijing-Tianjin-Hebei metropolitan region in China were recruited for this study. Participants were eligible for inclusion if they met the following criteria: (1) full-time employment in elderly care services; (2) a minimum of one year of work experience in the sector; and (3) voluntary participation with informed consent provided.

The survey instrument was developed through a systematic review of the domestic and international literature, complemented by field visits and focus group interviews conducted at multiple nursing homes to ensure contextual validity and practical relevance. The questionnaire consisted of two sections: Part I collected demographic and occupational characteristics, namely gender, age, educational attainment, monthly income, years of practice, and possession of professional certificates such as a registered nursing credential; Part II comprised validated scales to measure the core constructs of workplace social capital, occupational burnout, and turnover intention.

Prior to the formal survey, a pilot study was conducted with 30 caregivers from a nursing home in Beijing. Based on participant feedback, item wording was refined, and ambiguous or contextually inappropriate items were eliminated to enhance clarity and comprehension. The formal survey was then administered to caregivers in 15 nursing homes distributed throughout the provinces of Beijing, Tianjin, and Hebei.

Questionnaires were distributed electronically via QR codes generated on the Wenjuanxing, a widely used online survey system in China. Data collection was facilitated by junior undergraduate students majoring in Elderly Care Service and Management, who received specialized training prior to this fieldwork. The training program covered the research objectives of this study, its inclusion and exclusion criteria, ethical considerations, and standardized operational procedures. Before participation, the researchers provided all potential respondents with a comprehensive explanation of the study background, assured them of data anonymity and confidentiality, and emphasized the voluntary nature of their participation. Participants were also given instructions on proper questionnaire completion.

*A priori* power analysis was conducted using G*Power 3.1 (Faul et al., 2009) following the conventions established by Cohen (2013). With a medium effect size (*f*^2^ = 0.15), a significance level of *α* = 0.05, and a desired power (1–*β*) of 0.95, the required sample size was calculated. Given that the regression models included 14 predictors, the minimum required sample was 199. Our final sample (*N* = 222) exceeded this threshold, ensuring adequate statistical power.

A total of 384 questionnaires were initially collected. Following data screening, invalid responses were excluded based on two criteria: (1) completion time of less than 3 min, indicating insufficient attention to item content; and (2) evident patterned responses (e.g., straight-lining or alternating response patterns). After exclusion, 222 valid questionnaires remained, yielding an effective response rate of 57.8%. The final sample (*N* = 222) exceeded the minimum required sample size of 199, thereby ensuring adequate statistical power for the planned analyses.

The questionnaire comprised the following scales:

1. *Workplace Social Capital*. WSC was assessed using a scale adapted from Zhang et al. (2022) and revised to reflect the specific context of elderly care. This instrument comprises three dimensions: Bonding Social Capital (strong ties among peers), Linking Social Capital (vertical relationships with management), and Bridging Social Capital (cross-group connections). All items are rated on a 5-point Likert scale ranging from 1 = *strongly disagree* to 5 = *strongly agree*. Higher composite scores indicate higher levels of WSC. This independent variable is treated as continuous.
2. *Turnover Intention*. Turnover intention was assessed using a 4-item scale designed to capture tendencies toward both organizational and professional exit. All items are rated on a 5-point Likert scale ranging from 1 = *strongly disagree* to 5 = *strongly agree*, with total scores ranging from 4 to 20. Higher composite scores indicate stronger turnover intention. This dependent variable is treated as continuous.
3. *Occupational Burnout*. Occupational burnout was measured using a localized scale adapted from Jiang et al. (2019), based on Maslach’s burnout framework (Wang et al., 2019). Five representative items were selected to capture emotional exhaustion and depersonalization. All items are rated on a 7-point Likert scale ranging from 1 = *never* to 7 = *always*, yielding total scores ranging from 5 to 35. Higher composite scores indicate a higher level of occupational burnout. This variable is treated as continuous and it served as the mediator in the analytical model.
4. *Professional Certification Status*. This variable was operationalized as a dummy variable, with 0 = *absence of certificate* and 1 = *possession of certificate*. This variable served as the moderator in the analytical model.
5. *Control Variables*. Following the practice of prior studies, the following demographic and occupational characteristics were included as control variables: gender, age, educational attainment, monthly income, and years of practice. Gender was coded as a binary variable with *female* = 1 and *male* = 0 (reference). Age and years of practice were entered as continuous variables (in years). Their respective quadratic terms (Age^2^ and Years^2^) were also created to capture potential curvilinear effects. Theoretically, the relationship between age/tenure and turnover intention among nursing home caregivers may not be strictly linear: younger or newly hired caregivers may exhibit higher turnover intention due to job mismatch or low organizational commitment, whereas mid-career caregivers may become more settled, and older or very long-tenured caregivers may experience increased physical strain or burnout that elevates turnover intention again. Such U-shaped or inverted U-shaped patterns are well documented in occupational turnover research. Monthly income was measured using a 5-category ordinal variable: 2000–2,999 RMB, 3000–3,999 RMB, 4000–4,999 RMB, 5000–5,999 RMB, and >6,000 RMB.

### 3.2 Statistical analysis

Data analysis was conducted using SPSS 23.0 and AMOS 23.0, following a three-stage approach.

#### 3.2.1 Measurement model

Confirmatory factor analysis (CFA) was performed to evaluate the discriminant validity of the three latent constructs. Model fit was assessed using the following criteria: χ^2^/df < 3, CFI > 0.90, TLI > 0.90, RMSEA < 0.08, and SRMR < 0.08. All fit indices met the acceptable thresholds.

#### 3.2.2 Mediating analysis

Hierarchical linear regression was used to test the association and mediation pathways. All models controlled for the same set of covariates: gender, age (linear and quadratic terms), years of practice (linear and quadratic terms), monthly income (dummy variables, reference: 2,000–2,999 RMB), education (dummy variables, reference: high school or below), and professional certification (dummy: certified = 1). To reduce multicollinearity, age and years of practice were mean-centered prior to squaring (Aiken, 1991); WSC was also mean-centered. Four models were estimated sequentially: Model 1 included only covariates; Model 2 added WSC to test the association with turnover intention; Model 3 regressed occupational burnout on WSC; and Model 4 entered both WSC and occupational burnout.

All hierarchical linear regression models (Tables 1^2^–3) were estimated with heteroskedasticity-robust standard errors to address potential residual heteroskedasticity. By contrast, mediation and moderated mediation analyses (Tables 4–6) were implemented via Hayes’ PROCESS macro, which generates default non-robust standard errors paired with bias-corrected bootstrap confidence intervals. Although standard error calculation methods differ across analytical tools, the magnitude and statistical significance of all focal coefficients remain consistent.

**Table 1:** Regression analysis: Model 2.

Predictor	*B*	Robust standard error	*t*-statistic	Exact *P* > |t|	95% robust confidence interval	Variance inflation factor (VIF)
Gender (Female = 1)	−0.329	0.715	−0.46	0.646	[−1.738, 1.081]	1.12
age_c (Mean-centered age)	−0.003	0.048	−0.07	0.948	[−0.097, 0.091]	4.21
age_c2 (Mean-centered age squared)	0.002	0.003	0.67	0.505	[−0.003, 0.007]	2.39
year_c (Mean-centered years of practice)	0.239	0.106	2.27	0.024*	[0.031, 0.447]	3.44
year_c2 (Mean-centered years of practice squared)	−0.007	0.002	−2.83	0.005**	[−0.011, −0.002]	2.84
Monthly income: 3000–3,999 RMB	−0.257	1.182	−0.22	0.828	[−2.588, 2.074]	1.71
Monthly income: 4000–4,999 RMB	−1.297	0.891	−1.46	0.147	[−3.054, 0.460]	2.34
Monthly income: 5000–5,999 RMB	−2.368	0.979	−2.42	0.016*	[−4.297, −0.439]	2.47
Monthly income: >6,000 RMB	−1.550	1.193	−1.30	0.195	[−3.902, 0.802]	1.94
Education: College degree	2.305	1.409	1.64	0.103	[−0.472, 5.082]	3.61
Education: Bachelor’s degree	0.836	1.261	0.66	0.508	[−1.650, 3.321]	1.50
Professional certification (Yes = 1)	−1.286	0.739	−1.74	0.083	[−2.744, 0.171]	1.30
WSC_c (Mean-centered workplace social capital)	−0.250	0.062	−4.01	0.000***	[−0.373, −0.127]	1.18
Constant	1.315	1.038	1.27	0.206	[−0.731, 3.362]	
Model summary						
Number of observations (*N*)	222					
*R*-squared	0.296					
Overall model *F* statistic	7.21***					
Degrees of freedom (df)	13, 208					

Hierarchical model comparison for Model 1 vs. Model 2: Δ*R*^2^ = 0.094, Δ*F*(1, 208) = 16.08, *p* = 0.0001. Heteroskedasticity-robust standard errors are reported. All continuous variables were mean-centered. *B*, unstandardized coefficient. Robust SE reported. VIF, variance inflation factor. Income reference: 2,000–2,999 RMB. Education reference: high school or below. Gender: 0 = male, 1 = female. Certification: 0 = no, 1 = yes. *p < 0.05; **p < 0.01; ***p < 0.001.

**Table 2:** Regression analysis: Model 3.

Predictor	*B*	Robust standard error	*t*-statistic	Exact *P* > |*t*|	95% robust confidence interval	Variance inflation factor (VIF)
Gender (Female = 1)	−0.652	1.199	−0.54	0.587	[−3.015, 1.712]	1.12
age_c (Mean-centered age)	−0.073	0.079	−0.93	0.352	[−0.229, 0.082]	4.21
age_c2 (Mean-centered age squared)	−0.006	0.005	−1.20	0.233	[−0.016, 0.004]	2.39
year_c (Mean-centered years of practice)	0.309	0.172	1.80	0.074	[−0.030, 0.647]	3.44
year_c2 (Mean-centered years of practice squared)	−0.005	0.004	−1.35	0.178	[−0.013, 0.002]	2.84
Monthly income: 3000–3,999 RMB	−2.829	2.173	−1.30	0.194	[−7.113, 1.454]	1.71
Monthly income: 4000–4,999 RMB	−2.899	1.615	−1.80	0.074	[−6.082, 0.284]	2.34
Monthly income: 5000–5,999 RMB	−3.824	1.726	−2.22	0.028*	[−7.227, −0.421]	2.47
Monthly income: >6,000 RMB	−1.879	2.102	−0.89	0.372	[−6.024, 2.265]	1.94
Education: College degree	2.417	2.307	1.05	0.296	[−2.131, 6.964]	3.61
Education: Bachelor’s degree	−2.427	2.327	−1.04	0.298	[−7.015, 2.160]	1.50
Professional certification (Yes = 1)	−2.467	1.223	−2.02	0.045*	[−4.878, −0.055]	1.30
WSC_c (Mean-centered workplace social capital)	−0.250	0.107	−2.33	0.021*	[−0.461, −0.039]	1.18
Constant	4.885	1.942	2.52	0.013*	[1.057, 8.713]	—
Model summary						
Number of observations (N)	222					
*R*-squared	0.189					
Overall model *F* statistic	4.54***					
Degrees of freedom (df)	13, 208					

Heteroskedasticity-robust standard errors are reported. *B*, unstandardized regression coefficient; VIF, variance inflation factor. All continuous variables were mean-centered prior to analysis. Reference groups: monthly income = 2,000–2,999 RMB; education = high school or below. Gender was coded 0 = male, 1; certification was coded 0 = no, 1 = yes. *p < 0.05, ***p < 0.001.

**Table 3:** Regression analysis: Model 4.

Predictor	*B*	Robust standard error	*t*-statistic	Exact *P* > |*t*|	95% robust confidence interval	Variance inflation factor (VIF)
Gender (Female = 1)	−0.089	0.480	−0.19	0.852	[−1.035, 0.856]	1.12
age_c (Mean-centered age)	0.024	0.030	0.79	0.431	[−0.036, 0.083]	4.23
age_c2 (Mean-centered age squared)	0.004	0.002	1.92	0.057	[−0.000, 0.008]	2.41
year_c (Mean-centered years of practice)	0.126	0.074	1.71	0.089	[−0.020, 0.272]	3.51
year_c2 (Mean-centered years of practice squared)	−0.005	0.002	−2.92	0.004**	[−0.008, −0.002]	2.86
Monthly income: 3000–3,999 RMB	0.781	1.065	0.73	0.464	[−1.318, 2.881]	1.73
Monthly income: 4000–4,999 RMB	−0.233	0.618	−0.38	0.706	[−1.451, 0.985]	2.37
Monthly income: 5000–5,999 RMB	−0.965	0.717	−1.35	0.180	[−2.378, 0.449]	2.53
Monthly income: >6,000 RMB	−0.860	0.804	−1.07	0.286	[−2.444, 0.725]	1.95
Education: College degree	1.418	0.834	1.70	0.091	[−0.226, 3.062]	3.63
Education: Bachelor’s degree	1.726	0.708	2.44	0.016*	[0.331, 3.122]	1.51
Professional certification (Yes = 1)	−0.381	0.531	−0.72	0.474	[−1.428, 0.665]	1.33
WSC_c (Mean-centered workplace social capital)	−0.158	0.050	−3.14	0.002**	[−0.258, −0.059]	1.22
OB_c (Mean-centered occupational burnout)	0.367	0.042	8.64	0.000***	[0.283, 0.451]	1.23
Constant	−0.478	0.799	−0.60	0.551	[−2.054, 1.098]	—
Model summary						
Number of observations (N)	222					
*R*-squared	0.597					
Overall model *F* statistic	34.66***					

Heteroskedasticity-robust standard errors are reported. B, unstandardized regression coefficient; VIF, variance inflation factor. All continuous variables were mean-centered prior to analysis. Reference groups: monthly income = 2,000–2,999 RMB; education = high school or below. Gender was coded 0 = male, 1; certification was coded 0 = no, 1 = yes. *p < 0.05, **p < 0.01, ***p < 0.001.

**Table 4:** Decomposed cross-sectional associations.

Path	Path description	*B*	SE	*t*/Boot SE	*p*-value	95% CI
*a*	WSC_c → Occupational Burnout (M)	−0.2500	0.0846	−2.9537	0.0035	[−0.4168, −0.0831]
*b*	Occupational Burnout (M) → Turnover Intention (Y)	0.3670	0.0295	12.4273	<0.0001	[0.3088, 0.4253]
*C* (Total association)	WSC_c → Turnover Intention (no mediator)	−0.2500	0.0846	−2.9537	0.0035	[−0.4168, −0.0831]
*c′* (Direct association)	WSC_c → Turnover Intention (adjusted for M)	−0.1583	0.0368	−4.3011	<0.0001	[−0.2308, −0.0857]
*ab* (Indirect cross-sectional association)	WSC_c → OB_c → TI_c	−0.0917	0.0431	—	—	Bootstrap 95% CI [−0.1883, −0.0199]
Ratio of indirect to total association	Descriptive proportion statistic	0.367	—	—	—	—

All path coefficients and standard errors in this table are raw output from Hayes’ PROCESS Model 4, which adopts conventional non-robust standard errors; this differs from the heteroskedasticity-robust standard errors used in hierarchical regression Tables 1^2^–3. All substantive significance conclusions remain consistent under two estimation methods. The proportion of indirect cross-sectional association relative to total cross-sectional association was manually calculated. Covariates controlled in the model: gender, mean-centered age and its quadratic term (age_c/age_c2), mean-centered years of practice and its quadratic term (year_c/year_c2), income dummy variables (reference: 2,000–2,999 RMB), education dummy variables (reference: high school or below), professional certification dummy. Bias-corrected bootstrap confidence interval for indirect cross-sectional association was calculated based on 5,000 bootstrap resamples. An association is statistically significant if the 95% CI does not contain zero. *B*, unstandardized regression coefficient.

**Table 5:** Regression results for the first-stage moderated mediation model.

Variable	Stage 1: DV, occupational burnout (OB_c)				Stage 2: DV, turnover intention (TI_c)			
	*B*	*SE*	*t*	*p*	*B*	*SE*	*t*	*p*
Core predictors								
WSC_c	−0.554	0.163	−3.394	0.001	−0.161	0.037	−4.397	<0.001
WSC_c (conditional slope: certified caregivers)	−0.141	0.150	−0.94	0.348	—	—	—	—
OB_c	—	—	—	—	0.370	0.029	12.677	<0.001
Certification	−2.347	1.178	−1.993	0.048	—	—	—	—
WSC_c × certif	0.413	0.190	2.172	0.031	—	—	—	—
Covariates								
Edu_2 (college)	2.875	1.963	1.465	0.145	1.373	0.839	1.638	0.103
Edu_3 (bachelor’s)	−2.618	2.252	−1.163	0.246	1.787	0.965	1.851	0.066
Income2 (3000–3,999 RMB)	−2.110	2.029	−1.040	0.300	0.659	0.848	0.778	0.438
Income3 (4000–4,999 RMB)	−2.317	1.593	−1.455	0.147	−0.371	0.654	−0.567	0.571
Income4 (5000–5,999 RMB)	−3.286	1.782	−1.844	0.067	−1.130	0.733	−1.542	0.125
Income5 (>6,000 RMB)	−2.022	1.967	−1.028	0.305	−0.927	0.841	−1.102	0.272
Gender (female = 1)	−0.411	1.244	−0.331	0.741	−0.095	0.532	−0.177	0.859
Age_c	−0.088	0.074	−1.187	0.237	0.026	0.031	0.837	0.404
Age_c^2^	−0.007	0.005	−1.512	0.132	0.004	0.002	2.013	0.046
Year_c	0.317	0.142	2.229	0.027	0.118	0.061	1.946	0.053
Year_c^2^	−0.006	0.005	−1.318	0.189	−0.004	0.002	−2.299	0.023
Constant	4.274	1.933	2.211	0.028	−0.658	0.798	−0.825	0.411
Model fit								
*R* ^2^	0.207				0.596			
*F*	3.853*** (df1 = 14, df2 = 207)				23.556*** (df1 = 13, df2 = 208)			

*Path coefficients and standard errors in this table replicate raw output from Hayes’ PROCESS Model 4/7, which uses non-robust standard errors. This differs from the heteroskedasticity-robust standard errors adopted in the hierarchical regression tables (Tables 1^2^–3). ***p < 0.001. All coefficients are unstandardized B. Certification was coded as 0 = non-certified, 1 = certified. Income reference group: 2000–2,999 RMB. Education reference group: high school or below. All continuous predictors were mean-centered prior to analysis. The interaction term (WSC_c × certif) was constructed using mean-centered WSC. In the mediator model (left panel), the coefficient for WSC_c (−0.554) represents the simple slope for non-certified caregivers (certif = 0) in the presence of the interaction term, not the averaged main association reported in Table 12 Model 3. This is the standard interpretation of first-stage moderated mediation models (Hayes, 2013). For the conditional slope of certified caregivers (WSC_c + WSC_c × certif = −0.141), the standard error, t-statistic, and p-value were computed from the variance - covariance matrix of the interaction model. The 95% CI was calculated as −0.141 ± 1.96 × SE.

**Table 6:** Conditional indirect association and index of moderated mediation.

Association type	Group	Association value	Boot SE	95% bias-corrected bootstrap confidence interval
Conditional indirect association (WSC → OB → TI)	Non-certified caregivers (certif = 0)	−0.2052	0.0610	[−0.3400, −0.0973]
	Certified caregivers (certif = 1)	−0.0525	0.0493	[−0.1549, 0.0289]
Index of moderated mediation	Overall model	0.1527	0.0754	[0.0151, 0.3180]

Path coefficients and standard errors in this table replicate raw output from Hayes’ PROCESS Model 4/7, which uses non-robust standard errors. This differs from the heteroskedasticity-robust standard errors adopted in the hierarchical regression tables (Tables 1^2^–3). All bootstrap estimates are reported to four decimal places to exactly match the unrounded output in Supplementary File S1. Bootstrap resamples = 5,000. An association is statistically significant if the 95% confidence interval does not contain zero. The index of moderated mediation (0.1527) is calculated as the product of the WSC × certif interaction coefficient (*B* = 0.4126, see Table 5) and the burnout-to-turnover intention path (*B* = 0.3702). The index equals the difference between the conditional indirect association for certified and non-certified caregivers [−0.0525 − (−0.2052) = 0.1527], consistent with Hayes’ (2013) Model 7 specification. All associations are unstandardized coefficients.

#### 3.2.3 Moderated mediation

Mediation and moderated mediation were tested using Hayes’ PROCESS macro (Models 4 and 7) with 5,000 bootstrap resamples and 95% bias-corrected confidence intervals. The same covariate set was applied across all PROCESS models. Full PROCESS output is provided in Supplementary File S1.

#### 3.2.4 Diagnostic checks

Multicollinearity was assessed using variance inflation factors (VIFs), all of which ranged from 1.10 to 4.23, below the recommended cutoff of 5. Residual normality and homoscedasticity were confirmed via Q-Q plots and residual-versus-fitted plots. Heteroskedasticity-robust standard errors were adopted for all regressions. The Durbin–Watson statistic ranged from 1.707 to 2.087, indicating no significant autocorrelation.

## 4 Results

### 4.1 Demographics of the respondents

Table 7 presents the demographic and occupational characteristics of the 222 nursing home caregivers included in the final analysis. The sample was predominantly female (*n* = 176, 79.3%), with relatively few males (*n* = 46, 20.7%). For age distribution, participants above 50 years old constituted the largest proportion (*n* = 102, 45.9%), followed by those aged 30–50 years (*n* = 60, 27.0%) and aged 30 years and below (*n* = 60, 27.0%). Educational attainment varied across the sample, with 61.7% of caregivers holding a high school diploma or lower level of education (*n* = 137). With respect to work experience, 85.6% of participants reported 10 years or less of practice in the elderly care sector (*n* = 190). For monthly income, 63.5% of the caregivers earned less than 5,000 RMB per month (*n* = 141), while 36.5% reported a monthly income of 5,000 RMB or above (*n* = 81).

**Table 7:** Demographics of the respondents.

**Variables**	**Categories**	***N***	**Percentage**
Gender	Female	176	79.3%
	Male	46	20.7%
Age	Under 30 (included)	60	27.0%
	30–50 (included)	60	27.0%
	Above 50	102	45.9%
Education	High School and Below	137	61.7%
	College	69	31.1%
	Bachelor’s Degree	16	7.2%
Years of practice	≤ 5 Years	101	45.5%
	5–10 Years (included)	89	40.1%
	> 10 Years	32	14.4%
Monthly income (RMB)	2000–2,999	48	21.6%
	3,000–3,999	24	10.8%
	4,000–4,999	69	31.1%
	5,000–5,999	52	23.4%
	>6,000	29	13.1%
Professional certification	Yes	68	30.6%
	No	154	69.4%

### 4.2 Levels of occupational burnout and turnover intention

To characterize the distribution of occupational burnout and turnover intention among nursing home caregivers, we stratified burnout into three severity subgroups: low (5–10 points), moderate (11–20 points), and high (21–35 points). Turnover intention was likewise grouped into three tiers: low (4–10 points), moderate (11–15 points), and high (16–20 points). Table 8 displays the sample distribution of burnout severity. Specifically, 30.6% of participants reported low burnout (*n* = 68), 44.1% moderate burnout (*n* = 98), and 25.2% high burnout (*n* = 56). Collectively, nearly 70% of respondents (69.4%) experienced moderate to severe occupational burnout. Table 9 summarizes the distribution of turnover intention. A majority of caregivers (63.5%) demonstrated low turnover intention (*n* = 141), 27.5% reported moderate turnover intention (*n* = 61), and only 9.0% showed high turnover intention (*n* = 20). In aggregate, roughly one-third of the sample (36.5%) endorsed moderate to high turnover intention.

**Table 8:** Levels of occupational burnout (OB).

Variables	Categories	Low OB (*n* = 68)	Medium OB (*n* = 98)	High OB (*n* = 56)	Chi-Square	*p*
Gender	Female	55	78	43	0.324	0.850
	Male	13	20	13		
Age	Under 30 (included)	16	26	18	1.190	0.880
	30–50 (included)	19	27	14		
	Above 50	33	45	24		
Education	High School and Below	45	61	31	5.570	0.234
	College	16	30	23		
	Bachelor’s Degree	7	7	2		
Years of practice	≤ 5 Years	35	45	21	2.765	0.598
	5–10 Years (included)	23	40	26		
	>10 Years	10	13	9		
Monthly income (RMB)	2000–2,999	7	21	20	17.182	0.028
	3,000–3,999	7	13	4		
	4,000–4,999	24	31	14		
	5,000–5,999	22	22	8		
	>6,000	8	11	10		
Professional certification	Yes	12	32	24	9.523	0.009
	No	56	66	32		

**Table 9:** Levels of turnover intention (TI).

**Variables**	Categories	Low TI (*n* = 141)	Medium TI (*n* = 61)	High TI (*n* = 20)	Chi-Square	*p*
Gender	Female	115	43	18	4.713	0.095
	Male	26	18	2		
Age	Under 30 (included)	29	25	6	9.160	0.057
	30–50 (included)	41	14	5		
	Above 50	71	22	9		
Education	High School and Below	98	27	12	14.365	0.006
	College	32	30	7		
	Bachelor’s Degree	11	4	1		
Years of practice	≤ 5 Years	66	30	5	7.719	0.102
	5–10 Years (included)	52	27	10		
	> 10 Years	23	4	5		
Monthly income (RMB)	2000–2,999	18	24	6	28.733	0.000
	3,000–3,999	16	4	4		
	4,000–4,999	46	19	4		
	5,000–5,999	44	5	3		
	>6,000	17	9	3		
Professional certification	Yes	30	32	6	19.489	0.000
	No	111	29	14		

### 4.3 Reliability and validity assessment

Reliability was assessed using Cronbach’s *α* coefficient as the primary metric for evaluating internal consistency. The Cronbach’s α coefficients for all latent variables surpassed 0.90 (see Table 10), demonstrating the internal consistency of the measurement tools.

**Table 10:** Reliability and validity assessment.

Construct	Item	*B*	*β*	*Z*	*p*	Item reliability	CR	AVE
WSC	I feel that I am part of a whole with other caregivers	1.000	0.886		***	0.785	0.972	0.854
	Other caregivers and I care for each other	1.003	0.904	20.872	***	0.817		
	We trust our leaders	1.076	0.900	20.659	***	0.810		
	Leaders care about us	1.023	0.892	20.206	***	0.796		
	Colleagues inform each other when something happens	1.016	0.981	26.255	***	0.962		
	Colleagues help when encountering difficulties at work	1.018	0.977	25.929	***	0.955		
OB	Work makes me exhausted	1.000	0.869		***	0.755	0.941	0.762
	I feel very tired when I wake up in the morning	1.135	0.903	19.164	***	0.815		
	I feel a lot of work pressure	1.120	0.925	20.145	***	0.856		
	Working is driving me crazy	1.053	0.849	16.971	***	0.721		
	I am becoming less and less interested in my work	1.008	0.815	15.725	***	0.664		
TI	I have had thoughts of leaving this organization	1.000	0.860		***	0.740	0.939	0.794
	I feel there is no future for me here	1.047	0.882	17.771	***	0.778		
	I am also looking for other jobs	1.014	0.909	18.819	***	0.826		
	I do not want to be a nursing home caregiver any more	1.031	0.913	18.967	***	0.834		

*β*, standardized factor loading; *B*, unstandardized regression coefficient. ***p < 0.001.

Convergent validity was appraised using Composite Reliability (CR) and Average Variance Extracted (AVE). All standardized factor loadings were found to be ≥0.60, the CR values for all constructs were above 0.70, and the AVE values were consistently higher than 0.50, indicating satisfactory convergent validity.

Discriminant validity was assessed by comparing the square root of AVE for each latent variable against its correlations with other latent variables. As shown in Table 11, the square root of the AVE for each latent variable was consistently greater than its Pearson correlation coefficients with the remaining latent variables, providing compelling evidence for adequate discriminant validity.

**Table 11:** Discriminant validity.

Variable	WSC	OB	TI
WSC	**0.924**		
OB	−0.293**	**0.873**	
TI	−0.413**	0.709**	**0.891**

**p < 0.01. The bolded numbers represent the square roots of the AVE (Average Variance Extracted) corresponding to each construct.

### 4.4 Regression analysis

Table 12 presents the results of the hierarchical regression analysis. Model 1 incorporated all demographic control variables, including mean-centered linear and quadratic terms of years of practice to capture potential curvilinear effects. The joint significance test of the linear and quadratic tenure terms was significant (*F* = 8.06, *p* < 0.001), confirming a curvilinear association: the linear coefficient was positive (*B* = 0.334, *p* < 0.01), while the quadratic coefficient was negative (*B* = −0.008, *p* < 0.001). The theoretical inflection point was 20.875 centered years of practice, which falls within the observed tenure range. Although this threshold lies within the sample’s tenure distribution, only seven participants (3.2% of the total sample) reported centered years of practice exceeding this value. Accordingly, predicted turnover intention rose monotonically with tenure for the overwhelming majority of caregivers (96.8%). The downward tail of the fitted quadratic curve should be interpreted cautiously due to the extremely sparse observations in this high-tenure segment.

**Table 12:** Regression analysis: Model 1.

Predictor	*B*	Robust standard error	t statistic	Exact *P* > |*t*|	95% robust confidence interval	Variance inflation factor (VIF)
Gender (Female = 1)	0.125	0.678	0.19	0.853	[−1.210, 1.461]	1.10
age_c (Mean-centered age)	−0.024	0.051	−0.48	0.635	[−0.124, 0.076]	4.17
age_c2 (Mean-centered age squared)	0.000	0.003	0.02	0.983	[−0.006, 0.006]	2.36
year_c (Mean-centered years of practice)	0.334	0.097	3.46	0.001**	[0.143, 0.524]	3.27
year_c2 (Mean-centered years of practice squared)	−0.008	0.002	−3.87	<0.001***	[−0.012, −0.004]	2.80
Monthly income: 3000–3,999 RMB	−1.270	1.310	−0.97	0.333	[−3.852, 1.312]	1.66
Monthly income: 4000–4,999 RMB	−2.129	0.961	−2.22	0.028*	[−4.023, −0.236]	2.26
Monthly income: 5000–5,999 RMB	−3.516	1.023	−3.44	0.001**	[−5.533, −1.500]	2.35
Monthly income: >6,000 RMB	−2.878	1.218	−2.36	0.019*	[−5.278, −0.477]	1.84
Education: College degree	2.288	1.440	1.59	0.114	[−0.551, 5.127]	3.61
Education: Bachelor’s degree	0.844	1.441	0.59	0.559	[−1.996, 3.684]	1.50
Professional certification (Yes = 1)	−1.736	0.749	−2.32	0.021*	[−3.212, −0.259]	1.28
Constant	2.704	1.144	2.36	0.019*	[0.448, 4.960]	
Model summary						
Number of observations (N)	222					
*R*-squared	0.202					
Overall model *F* statistic	5.54***					
Degrees of freedom (df)	12, 209					
Mean VIF	2.35					

*B*, unstandardized coefficient. Robust SE reported. VIF, variance inflation factor. All continuous variables were mean-centered. Income reference: 2,000–2,999 RMB. Education reference: high school or below. Gender: 0 = male, 1 = female. Certification: 0 = no, 1 = yes. *p < 0.05; **p < 0.01; ***p < 0.001.

To validate the reliability of the quadratic tenure correlational pattern, we re-estimated the full covariate-adjusted regression specification (consistent with Model 1’s control set) with the addition of WSC after excluding the seven long-tenured participants with centered years of practice above the 20.875 inflection point, yielding a restricted analytical sample of *n* = 215. In this trimmed sample, the quadratic tenure term lost statistical significance (*B* = −0.002, *p* = 0.412), whereas the positive linear tenure association remained significant (*B* = 0.285, *p* = 0.002). Critically, the association between WSC and turnover intention remained nearly unchanged across the full and restricted samples: *B* = −0.271, *p* < 0.001 in the reduced sample versus *B* = −0.250, *p* < 0.001 in the original full sample. Full results for this robustness test are documented in Supplementary File S2.

**Figure 2:**
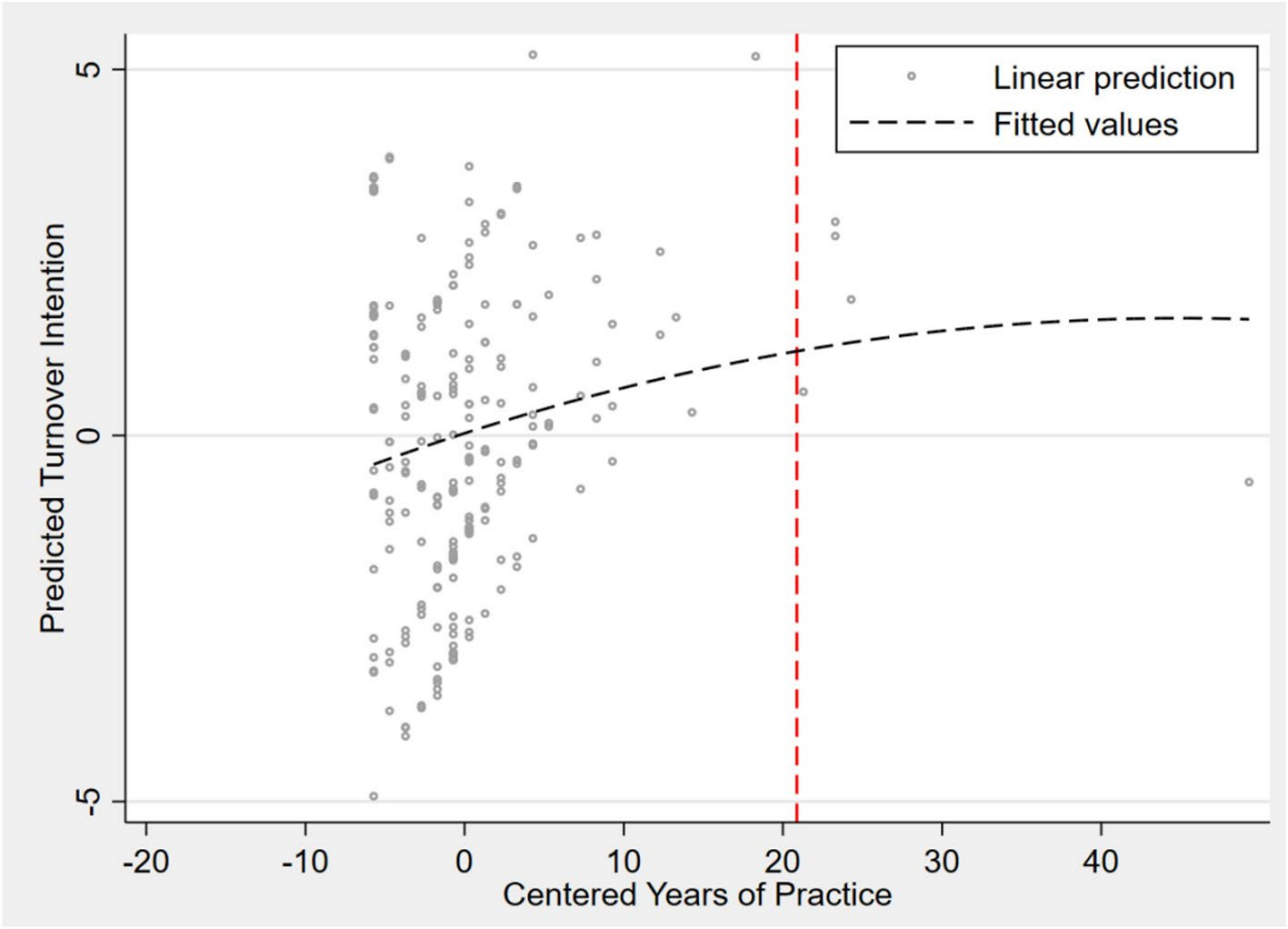
Predicted turnover intention across centered years of practice. The dashed black curve shows the inverted-U quadratic association between tenure and turnover intention, adjusted for all demographic covariates. The vertical red dashed line marks the theoretical turning point at 20.875 centered years of practice. Only seven participants (3.2% of the sample) fell beyond this threshold; for the remaining 96.8% of caregivers, predicted turnover intention rises monotonically with tenure. Scatter points represent model-adjusted predicted values. The descending tail of the curve should be interpreted cautiously, as it is based on only seven participants (3.2% of the sample). Robustness test results excluding the seven long-tenured outliers are reported in the text and Supplementary File S2.

Figure 2 visually demonstrates that the apparent inverted-U curvilinear pattern is driven almost exclusively by the seven long-tenured observations. For nearly all respondents, years of practice exhibited a stable positive linear relationship with turnover intention. Notably, the association between WSC and turnover intention remained virtually identical after removing these extreme high-tenure records, substantiating the robustness of our core Hypotheses H1–H3.

Monthly income was coded as a set of dummy variables, with the 2,000–2,999 RMB bracket serving as the reference group. Relative to this reference, the 4,000–4,999 RMB (*B* = −2.129, *p* < 0.05), 5,000–5,999 RMB (*B* = −3.516, *p* < 0.01), and >6,000 RMB (*B* = −2.878, *p* < 0.05) groups all exhibited significantly lower turnover intention. By contrast, the 3,000–3,999 RMB group did not differ significantly from the reference group. In summary, caregivers earning 4,000 RMB or above reported significantly weaker turnover intention than those in the 2,000–2,999 RMB bracket, while no significant difference was observed for the 3,000–3,999 RMB group.

As for age, the linear and quadratic age terms were non-significant across all regression models (all *p* > 0.05), and a joint F-test confirmed that the two coefficients were not jointly significant (*F* = 0.13, *p* = 0.882), indicating no curvilinear relationship between age and turnover intention in this sample.

To test Hypothesis 1, mean-centered WSC was added into Model 2 as the focal independent variable, raising the model *R*^2^ to 0.296 (*p* < 0.001), with an incremental explained variance of Δ*R*^2^ 0.094. An *F*-change test examining the unique contribution of WSC revealed Δ*F*(1, 208) = 16.08, *p* = 0.0001, indicating that WSC was negatively and significantly associated with turnover intention (*B* = −0.250, *p* < 0.001), supporting Hypothesis 1. Model 3 regressed occupational burnout on WSC. The results showed that WSC was significantly and negatively associated with occupational burnout (*B* = −0.250, *p* < 0.01).

Model 4 included both WSC and occupational burnout. WSC remained negatively associated with turnover intention (*B* = −0.158, *p* < 0.01), while occupational burnout showed a significant positive association (*B* = 0.367, *p* < 0.001). The coefficient for WSC decreased from −0.250 (Model 2) to −0.158 (Model 4), yet remained significant, suggesting that occupational burnout may partially mediate the relationship between WSC and turnover intention.

### 4.5 Mediating analysis

As shown in Table 4, WSC had a significant negative association with occupational burnout (path *a* = −0.2500, *p* < 0.01), and occupational burnout was positively and significantly associated with turnover intention (path *B* = 0.3670, *p* < 0.001). The total association of WSC with turnover intention was −0.2500 (*p* < 0.001), which decreased to *c’* = −0.158 after occupational burnout was added to the model as a mediator. The indirect association transmitted through occupational burnout was −0.0917, with a 95% bias-corrected bootstrap confidence interval of [−0.1883, −0.0199] that excluded zero. The estimated indirect association represented approximately 36.7% of the total statistical association in the specified cross-sectional model. Overall, the results support the hypothesized mediation structure by revealing a statistically significant indirect cross-sectional linkage from WSC to turnover intention via occupational burnout. Notably, the current study relies on cross-sectional data. These findings should be interpreted as statistical patterns rather than evidence of causal sequencing.

### 4.6 Moderated mediation analysis

To test the first-stage moderated mediation hypothesis (H3), Hayes’ PROCESS Model 7 was implemented with a consistent full set of demographic covariates identical to the hierarchical regression models in Table 12: mean-centered age and its quadratic term, mean-centered years of practice and its quadratic term, and dummy variables for education and monthly income. All continuous predictors including WSC were mean-centered prior to generating the interaction term of WSC × professional certification.

As presented in Table 5, the mediator regression predicting occupational burnout yielded a significant WSC × certification interaction term (*B* = 0.4126, *p* = 0.0310), confirming certification functions as a first-stage moderator of the WSC–burnout statistical association. The conditional simple slope of WSC predicting burnout for non-certified caregivers (certif = 0) was −0.5543 (SE = 0.1633, *t* = −3.3935, *p* = 0.0008), while the conditional slope for certified caregivers (certif = 1) attenuated to −0.1417 (SE = 0.1500, *t* = −0.9447, *p* = 0.3456). Critically, the mere contrast of significant versus non-significant slopes across subgroups cannot prove a meaningful group difference; the index of moderated mediation serves as the formal test for such discrepancy. The coefficient of WSC listed in the mediator column only represents the conditional association for non-certified caregivers under the interaction specification, which naturally differs from the average main association of WSC reported in Table 12 Model 3 without the cross-product term.

The corresponding bias-corrected bootstrap conditional indirect associations are reported in Table 6: the indirect pathway from WSC to turnover intention via burnout was statistically significant for non-certified caregivers (IE = −0.2052, 95% CI [−0.3400, −0.0973]) but non-significant for certified caregivers (IE = −0.0525, 95% CI [−0.1549, 0.0289]). This pattern indicates the negative indirect statistical linkage between WSC and turnover intention through burnout is weaker among certified caregivers.

Consistent with Hayes’ (2013) Model 7 specification, the index of moderated mediation is calculated as the product of the interaction coefficient for WSC × certif (*B* = 0.4126) and the path coefficient of occupational burnout predicting turnover intention (*B* = 0.3702), yielding an index value of 0.1527. The 95% bootstrap confidence interval for the index [0.0151, 0.3180] excluded zero, verifying a statistically meaningful difference in indirect associations between the two certification subgroups. This cross-sectional correlational pattern aligns with the theoretical expectation outlined in H3, though causal inference cannot be drawn given the single-time-point survey design.

In the outcome model predicting turnover intention (Stage 2 in Table 5), WSC retained a significant direct negative association with turnover intention after controlling for occupational burnout (*B* = −0.1609, *p* < 0.001). The full outcome regression explained 59.55% of the variance in turnover intention (*R*^2^ = 0.5955, *F* = 23.5564, df₁ = 13, df₂ = 208, *p* < 0.001). Table 5 fully reports both the mediator equation (DV = occupational burnout) and outcome equation (DV = turnover intention with PROCESS non-robust standard errors, distinct from heteroskedasticity-robust SEs in hierarchical regression tables), while Table 6 summarizes all conditional indirect associations and the index of moderated mediation based on 5,000 bootstrap resamples, with all values retained to four decimal places to exactly match raw output in Supplementary File S1.

## 5 Discussion

### 5.1 The association between workplace social capital and turnover intention

This study identified a significant negative correlational linkage between workplace social capital (WSC) and turnover intention among nursing home caregivers. WSC is a multidimensional construct capturing employees’ perceptions of workplace atmosphere, peer relationships and organizational norms, covering interpersonal trust, reciprocal support and collaborative communication. Unlike isolated single organizational factors, WSC serves as an integrated relational resource indicator, such that caregivers with higher perceived WSC tend to report lower turnover intention.

These correlational patterns align with prior empirical evidence. For example, Sarıgül and Uğurluoğlu (2023) documented negative associations between WSC and turnover intention mediated by organizational commitment and peer connections. Most existing relevant studies sampled hospital staff or decomposed WSC into separate fragmented dimensions, whereas this research specifically targets understudied nursing home caregivers who endure intense physical and emotional labor. The zero-order correlation between WSC and turnover intention was −0.411 (*p* < 0.001); the covariate-adjusted total association was −0.250 as displayed in Table 4. This raw correlation magnitude is relatively stronger than coefficients reported in hospital samples, implying that relational social resources carry greater weight in long-term care settings with limited worker autonomy and insufficient organizational support. Extending the work of Zhang et al. (2022) on Chinese social workers, this study shifts the research focus to elderly care staff and introduces professional certification as a first-stage moderator. By integrating Social Capital Theory and Conservation of Resources (COR) Theory, this paper further unpacks divergent resource buffering patterns across caregiver subgroups with distinct human capital credentials.

### 5.2 The mediating role of occupational burnout

Cross-sectional correlational analyses revealed a statistical indirect pathway from WSC to turnover intention via occupational burnout. Caregivers with abundant workplace social capital tend to receive consistent emotional and instrumental support from colleagues and supervisors, which correlates with lower burnout levels. In this covariate-adjusted model, burnout simultaneously co-varies with WSC and turnover intention, forming a statistical pattern consistent with mediation rather than a proven causal pathway. This pattern aligns with the core tenets of COR Theory, though single-time-point data cannot establish that improved WSC would causally reduce burnout symptoms.

This analysis advances prior research by jointly modeling the three focal variables instead of only reporting separate bivariate correlations (Zhang et al., 2022). The indirect statistical association accounted for approximately 36.7% of the covariate-adjusted total association between WSC and turnover intention; this proportion is purely descriptive and does not represent the causal explanatory power of burnout. This mediating pathway carries strong practical relevance for the long-term care sector, as 69.4% of participants reported moderate or severe burnout symptoms, highlighting the critical value of workplace relational resources in alleviating caregiver mental strain. Consistent with Barthauer et al. (2020), burnout exhibits robust concurrent correlation with turnover intention across care workforce cohorts, and the present joint regression model clarifies the tripartite correlational structure among WSC, burnout and turnover intention within this sample.

### 5.3 The moderating role of professional certification

Consistent with the first-stage moderated mediation results presented in Table 5, professional certification significantly moderated the statistical linkage between WSC and occupational burnout (the initial segment of the indirect pathway). Simple slope tests demonstrated a strong negative correlational linkage between WSC and burnout among non-certified caregivers (*B* = −0.554, *p* = 0.001), whereas this negative association was substantially weaker and non-significant for certified staff (*B* = −0.141, *p* = 0.348). It is inappropriate to judge subgroup differences merely by contrasting significant vs. non-significant slopes; the index of moderated mediation serves as the formal statistical test for discrepancies in indirect associations across groups. The index value was 0.1527 with a 95% bootstrap confidence interval [0.0151, 0.3180] the positive index of moderated mediation (Index = 0.1527), confirming that the magnitude of the indirect statistical association differs meaningfully by certification status. The non-significant indirect association among certified caregivers may stem partially from the smaller subgroup sample size (*n* = 68) and limited statistical precision, rather than a complete absence of the correlational pathway.

From the lens of COR Theory, professional certification represents stable personal human capital, and it correlates with a weaker negative concurrent linkage between WSC and burnout. Without credential-based professional advantages, non-certified caregivers lack intrinsic occupational resources; supportive workplace social capital therefore functions as a compensatory relational resource that correlates with reduced resource depletion and lower turnover intention. Unlike existing studies adopting psychological capital or organizational commitment as moderators, this paper selects policy-relevant professional certification as the first-stage boundary condition and provides subgroup association sizes to support differentiated human resource strategies for nursing home operators.

Notably, the positive index of moderated mediation (Index = 0.153) matches the calculation derived from Model 7 regression outputs. The index is computed by multiplying the coefficient of the WSC_c × certif interaction term (*B* = 0.413, see Table 5) by the burnout-to-turnover intention path coefficient (*B* = 0.370). A positive index does not contradict Hypothesis 3; it quantifies the gap between two negative indirect associations: the indirect correlational linkage is stronger for non-certified caregivers and attenuated for certified caregivers, which aligns with our theoretical prediction.

The sample was unbalanced across certification subgroups: 154 non-certified caregivers versus only 68 certified caregivers. The wider bootstrap confidence interval for the certified subgroup’s indirect association is largely attributable to insufficient subgroup statistical power, rather than a total disappearance of the correlational relationship. The index of moderated mediation offers a unified formal test of cross-group differences, independent of separate significance tests for each conditional indirect association.

### 5.4 Theoretical and practical contributions

This study offers two main theoretical contributions. First, it achieves a theoretically grounded integration of Social Capital Theory and COR Theory rather than a superficial juxtaposition of two frameworks. The cross-sectional statistical pattern supports an indirect correlational pathway from WSC to turnover intention via occupational burnout, enriching the resource depletion mechanism of turnover intention among long-term care workers.

Second, this research expands contingency perspectives in turnover research by identifying professional certification as a first-stage moderator. It clarifies that the strength of the WSC–burnout correlational linkage hinges on caregivers’ personal human capital endowments, refining contextual boundary conditions for the resource buffering function of workplace social capital.

Practically, all interpretations herein are limited to cross-sectional correlational patterns and cannot be treated as causal evidence for intervention effectiveness. Based on the observed statistical associations, we propose differentiated tentative management suggestions for nursing home administrators. For the majority of non-certified caregivers who display strong concurrent sensitivity to workplace relational environments, facilities offering regular team-building and peer mentorship programs appear more aligned with weaker turnover intention patterns observed in this cross-sectional sample. For certified caregivers, relational support alone shows weak concurrent correlation with retention; operators may combine interpersonal culture construction with tangible professional incentives, including certification allowances, standardized promotion pipelines and systematic vocational training. All proposed strategies are derived from single-wave survey data and require longitudinal intervention research to verify their actual efficacy.

## 6 Conclusion

This cross-sectional survey captures concurrent correlational patterns among variables, rather than definitive causal relationships. Based on a sample of 222 nursing home caregivers from the Beijing–Tianjin–Hebei region, this study constructed a first-stage moderated mediation model to unpack the interrelations between workplace social capital, occupational burnout, turnover intention and professional certification. WSC exhibited a significant negative covariate-adjusted correlation with turnover intention, and the joint distribution of the three core variables was consistent with an indirect pathway via burnout. Professional certification moderated the initial segment of this indirect linkage: the negative correlational chain from WSC to burnout and further to turnover intention was far more pronounced among non-certified caregivers, while the linkage remained weak and non-significant for certified staff with additional professional credential resources.

The documented correlational patterns provide tentative directional references for stabilizing the elderly care workforce amid China’s aging population. Nursing homes can adopt targeted retention strategies differentiated by certification background: prioritizing the cultivation of harmonious peer and managerial relationships for non-certified caregivers who rely heavily on workplace social resources; for certified staff, balancing relational support with formal professional rewards to lower turnover tendency.

Several limitations of this study must be acknowledged when interpreting the results, which also point out clear avenues for subsequent research. First, the cross-sectional research design prohibits causal inference. All variables were measured at a single time point, making it impossible to distinguish temporal sequences, eliminate reverse causality risks (e.g., high turnover intention may erode perceived workplace social capital), or rule out unmeasured confounding variables such as stable personality traits and institutional management systems. Longitudinal panel tracking or quasi-experimental designs are required to validate directional causal pathways proposed by the integrated theoretical framework. Second, the sampling scope is restricted to the Beijing–Tianjin–Hebei metropolitan area. Salary standards, pension policies, elderly care service supply and institutional operation modes vary widely across eastern coastal, central inland and rural western China, which restricts the national generalizability of our findings. Future investigations can adopt multi-region stratified sampling, including resource-limited rural nursing homes, to improve external validity. Third, the sample size satisfies statistical power to detect medium-to-large association magnitude as confirmed by the *a priori* power analysis, yet lacks sufficient precision for small association detection and fine-grained subgroup comparisons. The unbalanced distribution of certified (*n* = 68) and non-certified (*n* = 154) participants widened confidence intervals for the certified subgroup’s indirect association, reducing estimation accuracy. Follow-up research can expand the total sample and use stratified recruitment to balance subgroup sizes. Fourth, this study only examined professional certification as a first-stage moderator. Other potential boundary factors including psychological capital, work–family conflict and perceived organizational support remain underexplored. Subsequent research can incorporate multiple moderators to build a more complete nomological network of caregiver turnover intention. Bibliometric and systematic review analyses can also be conducted to map the overall intellectual landscape of this research field.

Despite the above constraints, this theory-driven empirical analysis delineates distinct resource buffering pathways of workplace social capital across caregiver subgroups, and offers differentiated targeted workforce management implications for China’s aging care sector.

## Data Availability

The original contributions presented in the study are included in the article/Supplementary material, further inquiries can be directed to the corresponding author.
