# Supplementary material for: Workplace social capital and turnover intention in nursing home caregivers: the mediating role of occupational burnout and the moderating role of professional certification
Source: Front Psychol. 2026 Jul 29;17:1822004. doi: 10.3389/fpsyg.2026.1822004 (PMC13461413; doi:10.3389/fpsyg.2026.1822004)
Supplement: Supplementary file 1 [file Table_1.DOCX]

**Supplementary File S1**

```
**************************************************************************
Model = 4
    Y = TI_c
    X = WSC_c
    M = OB_c

Statistical Controls:
CONTROL= edu_2    edu_3    income2  income3  income4  income5  gender   age_c    year_c   age_c2   year_c2  certif

Sample size
        222

**************************************************************************
Outcome: OB_c

Model Summary
          R       R-sq        MSE          F        df1        df2          p
      .4343      .1886    51.0760     3.7198    13.0000   208.0000      .0000

Model
              coeff         se          t          p       LLCI       ULCI
constant     4.8853     1.9296     2.5317      .0121     1.0811     8.6894
WSC_c        -.2500      .0846    -2.9537      .0035     -.4168     -.0831
edu_2        2.4169     1.9686     1.2277      .2209    -1.4641     6.2978
edu_3       -2.4273     2.2705    -1.0691      .2863    -6.9036     2.0489
income2     -2.8292     2.0196    -1.4008      .1628    -6.8107     1.1524
income3     -2.8992     1.5842    -1.8300      .0687    -6.0224      .2240
income4     -3.8241     1.7807    -2.1475      .0329    -7.3347     -.3135
income5     -1.8792     1.9835     -.9474      .3445    -5.7896     2.0312
gender       -.6516     1.2503     -.5212      .6028    -3.1165     1.8132
age_c        -.0734      .0741     -.9910      .3228     -.2194      .0726
year_c        .3086      .1433     2.1537      .0324      .0261      .5911
age_c2       -.0061      .0048    -1.2729      .2045     -.0155      .0033
year_c2      -.0051      .0045    -1.1259      .2615     -.0140      .0038
certif      -2.4668     1.1867    -2.0787      .0389    -4.8063     -.1272

**************************************************************************
Outcome: TI_c

Model Summary
          R       R-sq        MSE          F        df1        df2          p
      .7724      .5966     9.2673    21.8669    14.0000   207.0000      .0000

Model
              coeff         se          t          p       LLCI       ULCI
constant     -.4778      .8345     -.5725      .5676    -2.1230     1.1674
OB_c          .3670      .0295    12.4273      .0000      .3088      .4253
WSC_c        -.1583      .0368    -4.3011      .0000     -.2308     -.0857
edu_2        1.4178      .8416     1.6848      .0935     -.2413     3.0770
edu_3        1.7265      .9698     1.7802      .0765     -.1855     3.6384
income2       .7814      .8643      .9040      .3670     -.9226     2.4854
income3      -.2329      .6802     -.3424      .7324    -1.5740     1.1081
income4      -.9646      .7669    -1.2578      .2099    -2.4765      .5473
income5      -.8599      .8467    -1.0155      .3110    -2.5292      .8094
gender       -.0894      .5329     -.1678      .8669    -1.1400      .9612
age_c         .0238      .0316      .7527      .4525     -.0385      .0861
year_c        .1262      .0617     2.0447      .0422      .0045      .2478
age_c2        .0040      .0020     1.9464      .0530     -.0001      .0080
year_c2      -.0046      .0019    -2.3887      .0178     -.0084     -.0008
certif       -.3811      .5107     -.7462      .4564    -1.3880      .6258

******************** DIRECT AND INDIRECT EFFECTS *************************

Direct effect of X on Y
     Effect         SE          t          p       LLCI       ULCI
     -.1583      .0368    -4.3011      .0000     -.2308     -.0857

Indirect effect of X on Y
         Effect    Boot SE   BootLLCI   BootULCI
OB_c     -.0917      .0431     -.1883     -.0199

******************** ANALYSIS NOTES AND WARNINGS *************************

Number of bootstrap samples for bias corrected bootstrap confidence intervals:
     5000

Level of confidence for all confidence intervals in output:
    95.00
------ END MATRIX -----
**************************************************************************
Model = 7
    Y = TI_c
    X = WSC_c
    M = OB_c
    W = certif

Statistical Controls:
CONTROL= edu_2    edu_3    income2  income3  income4  income5  gender   age_c    year_c   age_c2   year_c2

Sample size
        222

**************************************************************************
Outcome: OB_c

Model Summary
          R       R-sq        MSE          F        df1        df2          p
      .4546      .2067    50.1797     3.8526    14.0000   207.0000      .0000

Model
              coeff         se          t          p       LLCI       ULCI
constant     4.2743     1.9332     2.2110      .0281      .4630     8.0856
WSC_c        -.5543      .1633    -3.3935      .0008     -.8764     -.2323
certif      -2.3466     1.1775    -1.9928      .0476    -4.6682     -.0251
int_1         .4126      .1900     2.1715      .0310      .0380      .7872
edu_2        2.8748     1.9626     1.4648      .1445     -.9945     6.7441
edu_3       -2.6182     2.2522    -1.1625      .2464    -7.0585     1.8220
income2     -2.1105     2.0290    -1.0402      .2995    -6.1107     1.8897
income3     -2.3171     1.5930    -1.4545      .1473    -5.4576      .8235
income4     -3.2863     1.7823    -1.8438      .0666    -6.8002      .2275
income5     -2.0221     1.9671    -1.0279      .3052    -5.9003     1.8561
gender       -.4114     1.2442     -.3306      .7413    -2.8643     2.0416
age_c        -.0875      .0737    -1.1868      .2367     -.2327      .0578
year_c        .3166      .1421     2.2287      .0269      .0365      .5967
age_c2       -.0072      .0047    -1.5124      .1320     -.0165      .0022
year_c2      -.0059      .0045    -1.3181      .1889     -.0148      .0029

Product terms key:

 int_1    WSC_c       X     certif

**************************************************************************
Outcome: TI_c

Model Summary
          R       R-sq        MSE          F        df1        df2          p
      .7717      .5955     9.2475    23.5564    13.0000   208.0000      .0000

Model
              coeff         se          t          p       LLCI       ULCI
constant     -.6580      .7980     -.8246      .4106    -2.2311      .9152
OB_c          .3702      .0292    12.6767      .0000      .3126      .4278
WSC_c        -.1609      .0366    -4.3973      .0000     -.2330     -.0888
edu_2        1.3731      .8385     1.6375      .1030     -.2800     3.0262
edu_3        1.7870      .9654     1.8510      .0656     -.1162     3.6901
income2       .6594      .8478      .7778      .4376    -1.0120     2.3308
income3      -.3708      .6539     -.5671      .5713    -1.6600      .9184
income4     -1.1305      .7332    -1.5420      .1246    -2.5759      .3148
income5      -.9271      .8410    -1.1024      .2716    -2.5851      .7309
gender       -.0945      .5323     -.1774      .8593    -1.1439      .9550
age_c         .0263      .0314      .8369      .4036     -.0356      .0882
year_c        .1181      .0607     1.9464      .0530     -.0015      .2378
age_c2        .0041      .0020     2.0125      .0455      .0001      .0081
year_c2      -.0044      .0019    -2.2989      .0225     -.0082     -.0006

******************** DIRECT AND INDIRECT EFFECTS *************************

Direct effect of X on Y
     Effect         SE          t          p       LLCI       ULCI
     -.1609      .0366    -4.3973      .0000     -.2330     -.0888

Conditional indirect effect(s) of X on Y at values of the moderator(s):

Mediator
         certif     Effect    Boot SE   BootLLCI   BootULCI
OB_c      .0000     -.2052      .0610     -.3400     -.0973
OB_c     1.0000     -.0525      .0493     -.1549      .0289

Values for quantitative moderators are the mean and plus/minus one SD from mean.
Values for dichotomous moderators are the two values of the moderator.

******************** INDEX OF MODERATED MEDIATION ************************

Mediator
          Index   SE(Boot)   BootLLCI   BootULCI
OB_c      .1527      .0754      .0151      .3180

When the moderator is dichotomous, this is a test of equality of the
conditional indirect effects in the two groups.

******************** ANALYSIS NOTES AND WARNINGS *************************

Number of bootstrap samples for bias corrected bootstrap confidence intervals:
     5000

Level of confidence for all confidence intervals in output:
    95.00

------ END MATRIX -----
```
