# Supplementary material for: Workplace social capital and turnover intention in nursing home caregivers: the mediating role of occupational burnout and the moderating role of professional certification
Source: Front Psychol. 2026 Jul 29;17:1822004. doi: 10.3389/fpsyg.2026.1822004 (PMC13461413; doi:10.3389/fpsyg.2026.1822004)
Supplement: Supplementary file 2 [file Table_2.DOCX]

**Supplementary File S2**

Robustness Check: Regression Model 1 (Excluding Seven Participants with Tenure Beyond the Turning Point (n = 215))

| Predictor | B | Robust SE | t | p | 95% CI | VIF |
| --- | --- | --- | --- | --- | --- | --- |
| Gender  (Female = 1) | 0.118 | 0.682 | 0.17 | 0.862 | [-1.224, 1.460] | 1.10 |
| age_c | -0.026 | 0.052 | -0.50 | 0.618 | [-0.128, 0.076] | 4.18 |
| age_c2 | 0.000 | 0.003 | 0.01 | 0.990 | [-0.006, 0.006] | 2.36 |
| year_c | 0.285 | 0.092 | 3.10 | 0.002 | [0.104, 0.466] | 3.15 |
| year_c2 | -0.002 | 0.003 | -0.82 | 0.412 | [-0.008, 0.003] | 2.68 |
| Monthly income: 3000–3999 RMB | -1.258 | 1.318 | -0.95 | 0.341 | [-3.856, 1.340] | 1.66 |
| Monthly income: 4000–4999 RMB | -2.118 | 0.968 | -2.19 | 0.030 | [-4.025, -0.211] | 2.26 |
| Monthly income: 5000–5999 RMB | -3.502 | 1.030 | -3.40 | 0.001 | [-5.533, -1.471] | 2.35 |
| Monthly income: > 6000 RMB | -2.865 | 1.226 | -2.34 | 0.020 | [-5.281, -0.449] | 1.84 |
| Education: College degree | 2.275 | 1.448 | 1.57 | 0.118 | [-0.580, 5.130] | 3.61 |
| Education: Bachelor's degree | 0.836 | 1.449 | 0.58 | 0.565 | [-2.021, 3.693] | 1.50 |
| Professional certification  (Yes = 1) | -1.728 | 0.753 | -2.29 | 0.023 | [-3.212, -0.244] | 1.28 |
| Constant | 2.698 | 1.151 | 2.34 | 0.020 | [0.428, 4.968] | — |

Model summary: R² = 0.1968, F(12, 202) = 5.12, p < 0.001. All coefficients are B with heteroskedasticity‑robust standard errors.

Robustness Check: Regression Model 2 (Excluding Seven Participants with Tenure Beyond the Turning Point (n = 215))

| Predictor | B | Robust SE | t | p | 95% CI | VIF |
| --- | --- | --- | --- | --- | --- | --- |
| Gender  (Female = 1) | -0.677 | 0.687 | -0.99 | 0.325 | [-2.031, 0.677] | 1.12 |
| age_c | 0.005 | 0.053 | 0.09 | 0.929 | [-0.100, 0.109] | 4.21 |
| age_c2 | 0.003 | 0.003 | 1.06 | 0.290 | [-0.002, 0.008] | 2.39 |
| year_c | 0.197 | 0.109 | 1.81 | 0.072 | [-0.018, 0.413] | 3.44 |
| year_c2 | 0.001 | 0.022 | 0.06 | 0.951 | [-0.042, 0.045] | 2.84 |
| Monthly income: 3000–3999 RMB | -0.359 | 1.236 | -0.29 | 0.772 | [-2.795, 2.077] | 1.71 |
| Monthly income: 4000–4999 RMB | -1.357 | 0.898 | -1.51 | 0.132 | [-3.128, 0.414] | 2.34 |
| Monthly income: 5000–5999 RMB | -2.391 | 0.967 | -2.47 | 0.014 | [-4.297, -0.485] | 2.47 |
| Monthly income: > 6000 RMB | -1.042 | 1.185 | -0.88 | 0.380 | [-3.379, 1.295] | 1.94 |
| Education: College degree | 1.836 | 1.396 | 1.31 | 0.190 | [-0.918, 4.589] | 3.61 |
| Education: Bachelor's degree | 0.792 | 1.341 | 0.59 | 0.555 | [-1.851, 3.435] | 1.50 |
| Professional certification  (Yes = 1) | -0.971 | 0.749 | -1.30 | 0.197 | [-2.448, 0.507] | 1.30 |
| WSC_c | -0.271 | 0.066 | -4.12 | <0.001 | [-0.400, -0.141] | 1.18 |
| Constant | 0.951 | 1.230 | 0.77 | 0.440 | [-1.475, 3.378] | — |

Note: Dependent variable = turnover intention (TI_c). Robust standard errors are reported. All continuous variables were mean-centered. Income reference group: 2,000–2,999 RMB. Education reference group: high school or below. WSC = workplace social capital. VIFs are computed from the OLS model without robust standard errors; mean VIF = 2.35. ***p < 0.001.
